# The impact of medical documentation assistants on process performance measures in a surgical emergency department

**DOI:** 10.1186/s40001-019-0390-9

**Published:** 2019-09-06

**Authors:** Johannes Lamprecht, Rainer Kolisch, Dominik Pförringer

**Affiliations:** 1 TUM School of Management, Technische Universität München, Arcisstr. 21, 80333 Munich, Germany; 2 Klinikum Rechts der Isar, Technische Universität München, Klinik und Poliklinik für Unfallchirurgie, Ismaningerstr. 22, 81675 Munich, Germany

**Keywords:** Medical documentation assistant, Surgical emergency department, Discrete event simulation, Process time, Waiting time

## Abstract

**Background:**

The administrative work of physicians, particularly documentation effort, consumes considerable time in surgical emergency departments. At the same time, the latter face an ever-growing influx of patients, leading to increasing waiting and flow times and thus patient dissatisfaction as well as overload of physicians and nurses. The deployment of medical documentation assistants, who specialize in and undertake documentation work currently performed by physicians, poses a solution to the problem. The goal of this study is to assess the impact of deploying medical documentation assistants on key performance indicators of a surgical emergency department, i.e. waiting and flow times of patients differentiated according to triage categories, utilization of physicians and time allocation of physicians.

**Methods:**

The underlying study has analysed the processes of the surgical emergency department of a major university medical centre and modelled them in a discrete event simulation. Data on patient arrivals as well as processing times in the X-ray department and the laboratory were obtained from the clinical information system, while processing times in the emergency department were recorded using time–motion studies. Though the emergency department currently does not deploy medical documentation assistants, the simulation model includes a variable number of such assistants.

**Results:**

The deployment of a medical documentation assistant frees up physician working time and decreases the waiting time and consequently the flow time of patients, in particular for standard and non-urgent patients. Adding additional documentation assistants leads to further improvements, however, with diminishing marginal returns. Under the assumption of medical documentation assistants being 35% more efficient than physicians in undertaking documentation work, one of the three physicians can be replaced in the analysed surgical emergency department with an average of 502 patient arrivals per week.

**Conclusions:**

Medical documentation assistants are a viable way of improving the performance of surgical emergency departments. Depending on the goals of the hospital, medical documentation assistants can be used for an array of measures such as decreasing patients’ waiting and flow times or increasing physicians’ time spent on medical treatment.

## Background

Two trends can be observed in German surgical emergency departments (ED): The number of patient arrivals continues to grow [1, 2], and the documentation work of physicians has risen continuously, leaving less time for direct patient care [3]. Both trends have led to an increase in waiting and flow times of patients and overcrowding of the surgical ED. One way to improve this situation is to deploy medical documentation assistants (MDAs). By taking over the documentation work from physicians, MDAs allow for a reallocation of capacity, leading to shorter patient waiting and flow times. Furthermore, physicians can spend a larger time share on medical work, such as direct patient care and research, providing higher satisfaction rates. Using discrete event simulation, we assess the impact of deploying MDAs in the surgical ED of a German university hospital. We assess the change in waiting and flow time for patients based on triage categories as well as time allocation and utilization of physicians. The underlying hypothesis of this research is that the presence of MDAs will accelerate the overall process, shorten waiting durations and treatment times in the benefit of patient and doctor.

## Data and methods

### Patient flow in the surgical ED and simulation model

The patient flow in the observed surgical ED can be depicted as follows: a patient enters the surgical ED, is registered at the reception and waits to be triaged by the triage nurse. Afterwards, he waits in the waiting area until called by a nurse. The nurse takes him into an examination room and examines him. When a physician becomes available, the nurse hands the patient over to the physician who performs a second examination. If necessary, the examination can include taking blood samples. After the physician’s examination, blood samples, if taken, are sent to the laboratory. Furthermore, if required, the patient is sent or transported to the X-ray department where an imaging procedure [X-ray or computed tomography (CT)] is conducted. Simultaneously, the physician undertakes the first part of the documentation. Once the results from the laboratory and/or the X-ray department have been received, the physician analyses them before consulting again with the patient to discuss the diagnosis and potential treatment. Following the treatment, the physician completes the documentation and hands it to the patient. If an in-hospital treatment becomes necessary, the physician transfers the patient to a specific clinic after organization of availability via phone. Whenever a physician cannot continue working with a patient due to the unavailability of the patient or of patient-related information (results of blood test, imaging), the physician starts treating a new patient. For patients, this results in an intermittent process where, whenever a physician is needed, patients potentially have to wait. During their shift, whenever time is available, physicians eat and fulfil personal needs.

The surgical ED and the patient flow have been modelled as a discrete event simulation using AnyLogic software version 8. Discrete event simulation is a well-established tool for assessing and improving the performance of EDs [4–6]. Physicians, the triage nurse as well as other nurses are modelled as resources with limited capacity. Though the surgical ED considered in this study currently does not deploy MDAs, the simulation model includes them as a resource. The availability of the physicians and the nurses follows the hospital shift schedules given in Table 1. MDAs are deployed in the day shift from 7:15 to 16:15. All simulation data reported below are related to the day shift.

**Table 1:** Physicians’ and nurses’ shift schedules

	16:00–08:15	07:15–16:15	14:00–23:00
Monday–Friday	1 Physician 3 Nurses	3 Physicians 4 Nurses	1 Physician 3 Nurses
Saturday, Sunday	1 Physician 3 Nurses	2 Physicians 4 Nurses	1 Physician 4 Nurses

Whenever documentation (initiation of the documentation, final documentation) has to be performed and an MDA is available, the task is carried out by the MDA, otherwise by the physician. Specifically, for the simulation without any MDA all documentation is done by the physicians. Documentation consists of writing the discharge letter as well as conduction of the so-called medical coding. Triage is conducted from 8:00 to 20:00; otherwise, patients are called on a first-come first-serve basis. The triage nurse, in accordance with the Manchester triage set of rules, classifies each patient into one of the following five categories [7]: immediate, very urgent, urgent, standard or non-urgent. Table 2 shows these categories and their maximum waiting time targets. While “immediate” patients are sent directly to the trauma room, all other patients are called according to minimum waiting time left until the maximum waiting time target is reached.

**Table 2:** Maximum waiting time targets for patient according to triage categories

Triage category	Immediate	Very urgent	Urgent	Standard	Non-urgent
Percentage	0.36	5.91	26.91	53.91	12.91
Maximum waiting time (min)	0	10	30	90	120

### Data collection

The data were retrieved from the clinical information system (CIS) for the time span from January to October 2017 and by conducting time–motion studies (TMS) in the surgical ED. A total of 23,000 patient arrivals from the CIS were analysed, and 1500 measurements of processing times were taken by time–motion studies from September through November 2017.

### Patient arrival

**Fig. 1:**
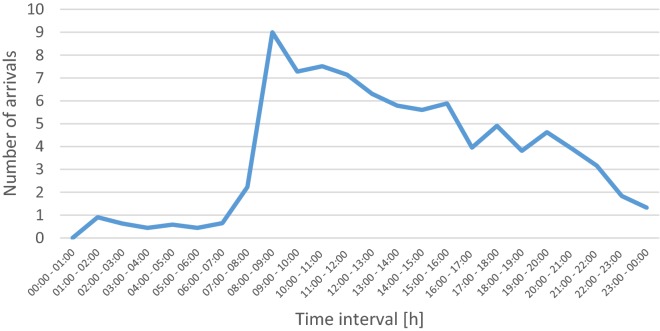
Average number of patients arriving per hour on Mondays

The patient arrival data retrieved from the CIS were statistically analysed. Applying goodness-of-fit tests using the Kolmogorov–Smirnov test for samples of size less or equal to 50 and the Chi-squared test otherwise, we determined the best fitting distributions for each hour of each day. The minimum, average and maximum numbers of patients arriving in an hour are 0, 3.31 and 18, respectively. The weekday with the most patients arriving is Monday, and the period with the most arriving patients is Monday from 8:00 to 9:00. Figure 1 shows the number of patient arrivals on Mondays.

### Process activities

Table 3 provides information about the resources required for each process activity applied to a patient, the mean and the standard deviation of the duration in minutes as well as the data source from which the processing time information was retrieved. Instead of fitting theoretical distributions, we used the empirical frequency distributions of the data in the simulation. For radiologic examination, the duration includes the travel time of the patient, the waiting time in the radiology department, the time for the imaging procedure and the time for sending the electronic images to the surgical ED. All radiologic imaging was seen by both trauma surgeon and radiologist. The time for processing a blood test is the time span from sending the blood samples via pneumatic tubes to the laboratory until the moment the results are made available in the clinical information system.

**Table 3:** Details of process activities

Process activity	Triage	Physical examination	Handing over patient (nurse to physician)	Physical examination and interviewing	X-ray	CT scan	Blood testing	Analysis of X-ray	Analysis of CT	Treatment (injection/cast/bandage etc.)	First documentation (of anamnesis and results)	Final documentation (completion of discharge letter)	Trauma room (urgent emergency)
Resource	Triage nurse	Nurse	Nurse, physician	Physician	X-ray department	X-ray department	Laboratory	Physician	Physician	Physician	Physician	Physician	Nurse, physician
Data source	TMS	TMS	TMS	TMS	CIS	CIS	CIS	TMS	TMS	TMS	TMS	TMS	TMS
Mean (time in min)	5.08	4.06	1.46	4.55	18.66	46.05	34.08	2.61	2.85	11.91	6.88	7.9	39.05
Standard deviation (in min)	3.73	3.09	1.58	2.85	16.67	32.05	–	2.31	1.76	11.21	5.3	5.47	13.08

## Results

**Fig. 2:**
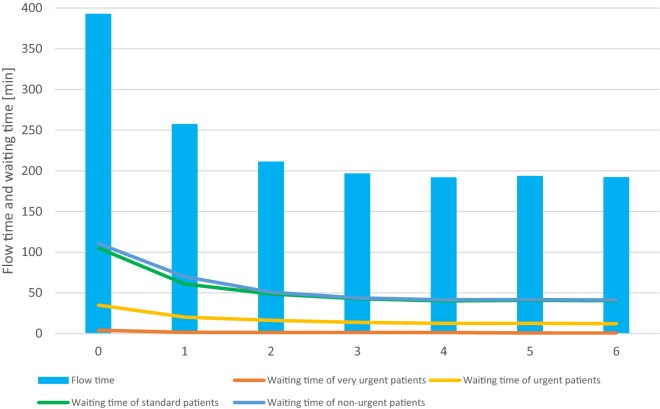
Average flow and waiting time of patients dependent on the number of MDAs

One week, undertaking 1000 replications was simulated. The average flow time of 392 min per patient as well as the average number of 502 patients per week reflects the actual data of the surgical ED. The average utilization of physicians and nurses is 78.9% and 53.0%, respectively. For the status quo, i.e. no deployment of MDAs, the first column of Fig. 2 shows the average flow time (over all patient categories) and the average waiting time of patients before being called by a nurse, differentiated by patient categories. Patients of all categories experience waiting times. However, while very urgent and urgent patients experience short waiting times, for standard and non-urgent patients the waiting times are considerable. Generally, waiting times increase with less urgent patient classes. Columns 2 to 6 in Fig. 2 give the impact of deploying different numbers of MDAs on flow time and waiting time. As shown, employing MDAs shortens the flow time, however, with decreasing marginal impact. The decrease in flow time is caused by a decrease in waiting times, in particular for the low-priority patients of categories “standard” and “non-urgent”. Deploying more than three MDAs in such cases does not further reduce waiting and flow times.

**Fig. 3:**
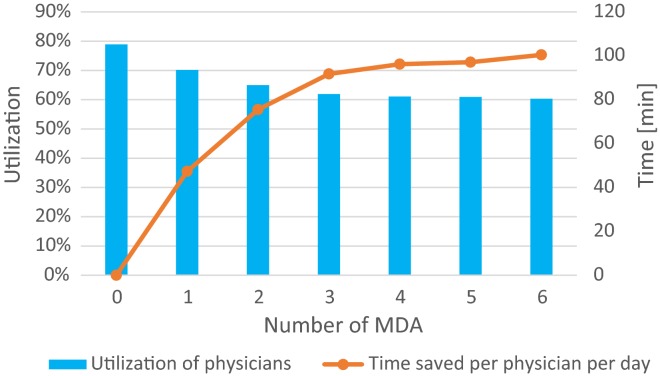
Utilization of physicians and daily time savings depending on the number of MDAs

**Fig. 4:**
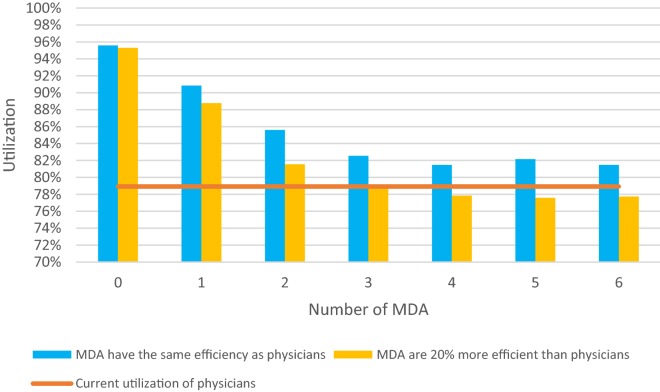
Substitution of one physician by MDAs

Figure 3 shows the effect of deploying MDAs on the utilization of physicians as well as the daily time saving per physician. Employing one MDA frees on average 47 min per physician per day. Figure 4 gives the average utilization of physicians for different numbers of MDAs if the number of physicians working during the day shift is reduced by one. Two specific cases are considered: first, if MDAs require the same time as the physicians to document cases and second, if the MDAs are 20% more efficient. Case 2 is based on the fact that MDAs are specialized in documentation work [8]. Figure 4 shows that without a higher efficiency one physician cannot be replaced by MDAs without increasing the utilization of the remaining physicians. However, for MDAs undertaking documentation 20% more efficiently than physicians, three MDAs can replace one physician without increasing the utilization of the remaining two physicians. Efficiency in this context was calculated in relation to the current time consumption for documentation work. As an example a 20% more efficiently working MDA would consume 20% less time for the same work.

Table 4 gives the average utilization of physicians during the day shift when two instead of three physicians are employed for different number of MDAs and different efficiency improvements of MDAs vs physicians. Italic values indicate that the resulting physician utilization is less than or equal to the current utilization of 78.9%. As can be seen, in the case of an improved MDA efficiency of 35% two MDAs can replace one physician without increasing the utilization of the remaining physicians.

**Table 4:** Utilization of two physicians depending on the number of MDA and their efficiency improvement. Average physician utilization in relation to number of MDAs and varying efficiency improvements

Improvement of documentation efficiency/number of documentation assistants (%)	0	1	2	3	4	5	6
0	0.95586138	0.90851259	0.85600901	0.82543679	0.81478161	0.8216141	0.81484681
5	0.95116114	0.90154917	0.84816247	0.81656295	0.80537848	0.80877604	0.8058309
10	0.95644712	0.89779539	0.83217917	0.81203976	0.80236947	0.79904161	0.7951842
15	0.95450305	0.89039862	0.82852664	0.7962661	0.79050967	*0.78706467*	*0.78376729*
20	0.95310867	0.8877983	0.81547893	*0.78853803*	*0.77847204*	*0.77588683*	*0.77737212*
25	0.95508084	0.87670289	0.80305884	*0.77843705*	*0.77023271*	*0.76783064*	*0.76252425*
30	0.95138378	0.87609568	0.79106149	*0.76401855*	*0.75805195*	*0.74755924*	*0.75663769*
35	0.95539147	0.86085714	*0.78118526*	*0.75060058*	*0.74887092*	*0.74933448*	*0.75064856*
40	0.9563154	0.84815266	*0.7646406*	*0.73999072*	*0.73382242*	*0.73223801*	*0.73101034*
45	0.95517466	0.84099205	*0.74957196*	*0.72704585*	*0.72341399*	*0.72533766*	*0.72312589*
50	0.956392	0.82909488	*0.74157911*	*0.71411821*	*0.71506467*	*0.71098648*	*0.71082958*

Italic values are significant at 0.05 probability level

## Discussion

Physicians are spending more time on documentation work which leads to spending less time on direct patient care [3]. In a survey study by Oxentenko et al. [9] undertaken in the USA in 2010, 67.9% of the 16,402 responding internal medicine residents assert that they spend more than 4 h per day on documentation. Using a work sampling approach, conducted in the ED of a Danish hospital, Füchtbauer et al. [10] report physicians spent 30% of the time on direct patient care and 31% on documentation. Analysing the measurements of 35 observers who witnessed over 162 h in which 439 consultations were performed by in total 24 physicians of two different university hospitals in the Netherlands, Joukes et al. [11] state an average of 31% and 26% of time spent on documentation for hospitals one and two, respectively. Undertaking a time–motion study in an Australian teaching hospital with 6.243 recorded physician tasks, Westbrook et al. [17] measured, amongst other tasks, the percentage of documentation work, excluding medication documentation. On average, doctors spend 14% of their time on documentation. However, the percentage differs significantly between the groups of doctors. While registrars and residents spend only 7.1% and 11% of their time on documentation, interns spend with 22% almost twice as much on documentation. Ammenwerth and Spötl [12] undertook a work sampling analysis with 5555 observations in a Austrian hospital with a precise categorization of tasks distinguishing between and further detailing clinical documentation (e.g. documentation of findings, writing of discharge letters) and administrative documentation (e.g. diagnosis coding). They report that 27.5% of the physicians’ time was spent on direct patient care, 22.4% on clinical documentation and 4.2% on administrative documentation. Perry et al. [18] confirm these findings by conducting a time–motion study that is based on more than 40 h of observation. Looking at ED physicians’ time, their results state a share of 21.6% and 2.4% for paperwork and administrative tasks, respectively. Tipping et al. [13] analysed a total of 494 h of observation conducted in 24 different hospitals in the USA. Performing the documentation task using an electronic medical records system takes on average 34.1% of a physician’s daily available time. In the survey of Blum and Müller [14] based on the responses of 1010 physicians in German hospitals, clinical documentation accounts for 25.08% and 32.46% of physician’s working time in surgical clinics and internal medicine clinics, respectively. Undertaking a time–motion analysis that builds on in total 4.250 observed activities performed by surgeons and internists of a municipal 300-bed teaching hospital in Southern Germany, Weigl et al. [19] measure, amongst other tasks, documentation and activity documentation (DRG coding). Their results show that surgeons and internists spend 25.7% and 32% of their time on documentation, respectively. This confirms the findings of Blum and Müller [14].

Our study, undertaken in the surgical ED of a German university hospital, shows 21.54% of the physician’s time is spent on documentation, which is comparable to the data obtained by Perry et al. [18] as well as Ammenwerth and Spötl [12].

By deploying MDAs, physicians can be freed from clinical and administrative documentation work. Haack [15] reports on employing MDAs with a focus on clinical coding in two German clinics. The results show that MDAs improve the quality of coding and thus have a positive economic impact. Schimdt et al. [16] present a pilot study for employing an MDA in a German trauma surgery and, like Haack, conclude a positive economic impact due to enhanced clinical coding. Linczak et al. [8] report the deployment of an MDA in a German clinic for traumatology and reconstructive surgery. Based on the lower costs of MDAs compared to physicians, they calculate that 2.5 MDAs can be financed per 5000 cases per year. In contrast to the studies of Haack and Schmidt et al., our study assesses the impact of deploying MDAs for clinical documentation rather than clinical coding. Furthermore, we assess the impact of MDAs with a stochastic–dynamic discrete event simulation study rather than with a static comparison on a cost per documentation basis. Our findings show that employing MDAs considerably reduces waiting and flow times of patients, in particular of standard and non-urgent patients. Furthermore, we show that for a surgical ED treating on average 502 patients per week, staffed with three physicians and four nurses working during the day shift, employing one MDA can allow for a reallocation of up to 47 min per physician per day, time which can be used for more direct patient care or, in the case of university hospitals, for research. If one MDA can substitute for a full physician role by time being saved, the income gap between the two roles is available for additional patient services or can be invested into technology or research.

## Conclusions

Deploying MDAs in a surgical ED reduces waiting time and flow time of patients, in particular of patients with low triage priority. Furthermore, deploying one MDA can free up 47 min per physician per day, which can be used for more direct patient care.

## Limitations

One significant limitation of this research is the fact that only one hospital was examined.

## Data Availability

The datasets generated during and/or analysed during the current study are available from the corresponding author, Dominik Pförringer, upon reasonable request.

## Abbreviations

CIS: clinical information system
CT: computed tomography
ED: emergency department
MDA: medical documentation assistant
TMS: time–motion studies
